# Mycobacterial immune reconstitution inflammatory syndrome in HIV-1 infection after antiretroviral therapy is associated with deregulated specific T-cell responses: Beneficial effect of IL-2 and GM-CSF immunotherapy

**DOI:** 10.1186/1476-8518-3-7

**Published:** 2005-09-25

**Authors:** A Pires, M Nelson, AL Pozniak, M Fisher, B Gazzard, F Gotch, N Imami

**Affiliations:** 1 Department of Immunology Imperial College London, Chelsea and Westminster Hospital, 369 Fulham Road, London. UK; 2 Department of HIV/GU Medicine, Chelsea and Westminster Hospital, 369 Fulham Road, London, UK; 3 Department of HIV/GU Medicine, Royal Sussex County Hospital, Brighton, UK

**Keywords:** Immune reconstitution, T cells, HIV-1, Mycobacterial infection, MAC

## Abstract

**Background:**

With the advent of antiretroviral therapy (ART) cases of immune reconstitution inflammatory syndrome (IRIS) have increasingly been reported. IRIS usually occurs in individuals with a rapidly rising CD4 T-cell count or percentage upon initiation of ART, who develop a deregulated immune response to infection with or without reactivation of opportunistic organisms. Here, we evaluated rises in absolute CD4 T-cells, and specific CD4 T-cell responses in 4 HIV-1^+ ^individuals presenting with mycobacterial associated IRIS who received in conjunction with ART, IL-2 plus GM-CSF immunotherapy.

**Methods:**

We assessed CD4 T-cell counts, HIV-1 RNA loads, phenotype for naïve and activation markers, and *in vitro *proliferative responses. Results were compared with those observed in 11 matched, successfully treated asymptomatic clinical progressors (CP) with no evidence of opportunistic infections, and uninfected controls.

**Results:**

Median CD4 T-cell counts in IRIS patients rose from 22 cells/μl before initiation of ART, to 70 cells/μl after 8 months of therapy (median 6.5 fold increase). This coincided with IRIS diagnosis, lower levels of naïve CD4 T-cells, increased expression of immune activation markers, and weak CD4 T-cell responses. In contrast, CP had a median CD4 T-cell counts of 76 cells/μl at baseline, which rose to 249 cells/μl 6 months post ART, when strong T-cell responses were seen in > 80% of patients. Higher levels of expression of immune activation markers were seen in IRIS patients compared to CP and UC (IRIS > CP > UC). Immunotherapy with IL-2 and GM-CSF paralleled clinical recovery.

**Conclusion:**

These data suggest that mycobacterial IRIS is associated with inadequate immune reconstitution rather than vigorous specific T-cell responses, and concomitant administration of IL-2 and GM-CSF immunotherapy with effective ART may correct/augment T-cell immunity in such setting resulting in clinical benefit.

## Background

The degree of immune reconstitution observed in HIV-1^+ ^individuals following initiation of antiretroviral therapy (ART), is variable [1-4]. Although seen even in late-stage disease, it is more prominent in patients who commence treatment during early HIV-1 infection before substantial damage to the immune system, where robust responses are often seen after treatment [5-7]. Such responses likely reflect effective immune surveillance, mimicking the beneficial T-cell responses seen in untreated long-term non-progressors, HIV-1 exposed but seronegative individuals, and after therapeutic vaccination of asymptomatic patients [8-10].

It has been postulated that after treatment of late-stage HIV-1 infection, recovery and augmentation of immune function, and responses to previous sub-clinical infections with existing pathogens such as *Mycobacterium spp*, hepatitis B and hepatitis C viruses, or cytomegalovirus (CMV) may result in exacerbated inflammatory diseases [11-19]. This phenomenon, described by others as immune reconstitution inflammatory syndrome (IRIS), is mostly seen in profoundly immunosuppressed patients with CD4 T-cell nadirs of less than 100 cells/μl, who upon receiving ART rapidly achieve an undetectable plasma viremia, and experience a very rapid increase in CD4 T cells [12,14]. This complex syndrome presents with either active opportunistic infections, or recurrence of previous infections.

We investigated the quality and breadth of lymphoproliferative responses in a group of HIV-1^+ ^patients on stable ART for > 6 months with suppressed viremia, diagnosed with *Mycobacterium avium *complex (MAC) associated IRIS, who were unresponsive to conventional anti-MAC therapy. We showed that these patients lacked pathogen-specific *in vitro *T-cell responses suggesting that the degree and quality of immune reconstitution following ART is inadequate to eliminate underlying opportunistic infections. Furthermore, immunotherapy with IL-2 and GM-CSF in combination with effective ART appears to accelerate augmentation of specific CD4 T-cell responses and increase the rapidity of immune recovery allowing underlying opportunistic infections to be cleared and leading to a better immediate outcome and resolution of IRIS.

## Methods

### Subjects studied

Fifteen HIV-1^+ ^patients at the Chelsea and Westminster Hospital, London, UK were studied. Four presented with MAC-associated IRIS, and where acid-fast bacilli were detected, patients were given anti-MAC therapy as soon as diagnosed. These patients had a median CD4 T-cell count of 22 cells/μl (interquartile range (IQR) 6.3–50.3) before initiation of ART, rising to 70 cells/μl (IQR 63–123) after 8 months of therapy (Table 1). For clarity these subjects will be referred to as IRIS patients. Previous reports define IRIS as a syndrome occurring in individuals with a rising CD4 T-cell count or percentage upon initiation of ART, who develop new clinical pathologies with either a new clinical presentation or reactivation of opportunistic organisms [15,16]. Viral load was undetectable in all patients at presentation of IRIS. IRIS patients (n = 4) received immunotherapy as salvage therapy consisting of IL-2 (Chiron Therapeutics, Uxbridge, UK) at 5 million units twice daily subcutaneously for 5 days, in three cycles 4 weeks apart. During the third cycle of IL-2, concomitant GM-CSF (Novartis, Schering-Plough, Camberley, UK) was administered subcutaneously 150 μg daily for 5 days. The remaining patients were asymptomatic clinical progressors (n = 11) receiving ART for 6 months, with a median CD4^+ ^T-cell count of 76 cells/μl (IQR 22.5–90) at baseline, rising to 249 cells/μl (IQR 187.5–303.5) 6 months post ART, and with viral load levels from undetectable (80% of patients) to 127 HIV-1 RNA copies/ml plasma. These patients developed no secondary effects following treatment, and had no evidence of opportunistic infections/exacerbated immune responses. Sixteen healthy HIV uninfected donors were used as controls. Informed consent was obtained from all patients for the administration of immunotherapy and investigations carried out, and ethics committee approval was obtained for the studies described.

**Table 1:** Clinical features of IRIS patients

Patient	CD4 T cell count before ART cells/μl	CD4 T cell count at presentation of IRIS cells/μl	Fold change in CD4 T cell counts from baseline to IRIS presentation	CD4 T cell count after remission of IRIS cells/μl	HIV-1 RNA at presentation of IRIS copies/ml	Reason for admission	Time on therapy*	Therapy
1	7	69	9.86	202	U/D	MAC	8	d4T+ddI+NFV+ImRx
2	37	70	1.89	140	U/D	MAC	12	AZT+3TC+IDV+ImRx
3	4	45	11.25	93	U/D	MAC	18	d4T+ddI+NFV+ImRx
4	90	280	3.11	601	U/D	MAC	8	AZT+3TC+EFV+ImRx

*Time in months from initiation of potent ART until diagnosis of IRIS. Drugs used in ART regime: Nucleoside analogues; Stavudine (d4T), Didanosine (ddI), Lamivudine (3TC) and Zidovudine (AZT) Protease inhibitors; Nelfinavir (NFV), Indinavir (IDV), or Non-nucleoside reverse transcriptase inhibitor; Efavirenz (EFV); ImRx = immunotherapy; MAC = *Mycobacterium avium *complex.

### Plasma viral RNA assay

Viral load was measured at each time point of sample collection using the Bayer HIV-1 RNA 3.0 assay (bDNA) (Bayer Diagnostics, Newbury, UK) with lower detection limit of 50 HIV-1 RNA copies/ml plasma.

### Lymphocyte subset quantification

The Epics XL-MCL (Beckman Coulter, High Wycombe, UK) was used for four-colour flow cytometric analysis. Anti-human CD3, CD4, CD8, and CD45 were used to analyze T cell subsets. Leukocytes were analysed on the Epics XL-MCL flow cytometer using system II software in conjunction with control reagents (Beckman Coulter) which provide automated colour compensation, light scatter and colour intensities.

### T cell proliferation assay

Peripheral blood mononuclear cells (PBMC) were cultured in triplicate with HIV-1 or other recall/viral antigen in round-bottomed microtiter plates (Greiner, Gloucester, UK) for 5 days as described previously [20-22]. The antigens used were: Herpes simplex virus (HSV), purified avian protein derivative of tuberculin (PPD), tetanus toxoid (TTox), Varicella-Zoster virus (VZV), Candida (CAN), and Cytomegalovirus (CMV) as described in reference 21. HIV-1 recombinant antigens were obtained from the Medical Research Council Centralised Facility for AIDS Reagents (National Institute for Biological Standards and Controls, Potters Bar, UK) and comprised: recombinant HIV-1-nef, recombinant HIV-1-gp120 and recombinant HIV-1-p24 (all used at 10 μg/ml final concentration) [22]. Adjuvant-free Remune and its native-p24 antigens were a generous gift from Dr Ronald Moss (Immune Response Corporation, Carlsbad, USA) and were used at 3 μg/ml to ensure that anti-HIV-1 responses were not overlooked due to clade variability. On day 5, 100 μl of supernatant was collected from each well and stored at -20°C for subsequent cytokine measurement, cells were pulsed with [^3^H]thymidine (Amersham International, Amersham, UK) and 16 h later cells were harvested onto glass fiber filtermats and counted (Wallac Oy, Turku, Finland). Results are expressed as stimulation indices (SI) with a positive response defined as an SI of 3 or more and Δ counts per minute (CPM) > 600 as described previously [21-23]. Control wells, for calculation of background activity, contained PBMC only.

### Measurement of IL-4 production

Fifty μl of supernatant from proliferative cultures was transferred to 96-well round-bottomed plates in triplicate for quantification of IL-4 on the indicator cell line CT.h4S (a generous gift of W. Paul, Bethesda, MD) as previously described [20-22]. Briefly, CT.h4S (5 × 10^3 ^cells/well), were added in 50 μl to 50 μl of supernatant to give a final volume of 100 μl. After 24 h in culture, wells were pulsed with [*methyl*-3H]thymidine, and cells were harvested as described above. Results are expressed as the mean cpm for triplicate cultures, with an error of the mean of ± 15%. A positive result is defined as significant proliferation above the background activity and detection threshold. In all experiments, a standard titration of indicator cell proliferation to a range of recombinant IL-4 from 0.01 to 100 U/ml was included. Control wells for calculation of background activity contained indicator cells only.

### Phenotypic analysis of lymphocytes

PMBC were incubated with a panel of murine anti-human mAbs (all Beckman Coulter), for 30 minutes at 4°C. Directly conjugated antibodies used were: Fluorescein isothiocyanate (FITC)-CD8, CD45RA; Phycoerytherin (PE)-CD38, HLA-DR, CD27, and CD45RA; PE-cyanine (PC-5)-CD4, all used according to the manufacturer's instructions. Cells were washed and fixed in PBS containing 2% paraformaldehyde (Sigma). On acquisition, a gate was set around the lymphocyte population on a forward scatter versus side scatter dot plot, and 10,000 gated events collected for each sample. Data analysis was performed using CELLQuest™ Software (Becton Dickinson, Oxford, UK). Appropriate isotype matched controls were run in parallel for each sample.

### Statistical analysis

Computer software (Statview 5.01; Abacus, Berkeley, CA) was used for all statistical calculations. Data are presented as median (inter-quartile range IQR). Analysis of data between the different groups was performed using a Mann Whitney-U-test and intra-group variations were compared using the Wilcoxon signed rank test. *P *values below 0.05 were considered significant.

## Results

### Patients

We studied both IRIS and CP patients who had been receiving effective ART for similar periods. IRIS was diagnosed at a median 10 months (IQR 8–13) after initiation of ART (Table 1). This is in agreement with previous reports [24]. The patients did not recover from the underlying MAC infection despite receiving conventional anti-MAC treatment, and were given IL-2 and GM-CSF in conjunction with ART as salvage therapy as detailed in materials and methods and as previously described [9,21]. Increases in CD4 T-cell counts from baseline to IRIS diagnosis were observed in all patients. Viral load reached BDL in all patients and remained undetectable throughout the study.

### IRIS patients receiving ART plus immunotherapy

**Figure 1:**
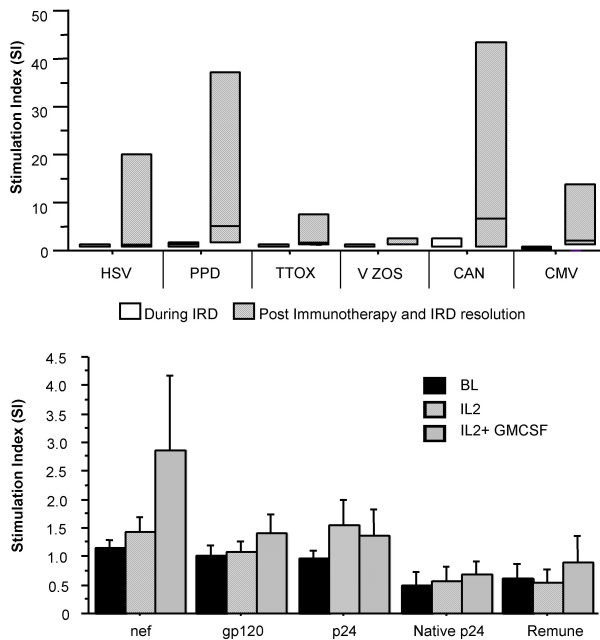
**(a – top) Lymphoproliferative responses to a panel of recall antigens in IRIS patients during IRIS manifestation and post remission. **Open bars denote T cell responses during IRIS manifestation and hatched bars represent T cell responses after immunotherapy with IL-2 plus GM-CSF in conjunction with ART and resolution of IRIS. Data are shown as median SI values with interquartile ranges. X-axis depicts the recall antigens tested. **(b- bottom) HIV-1-specific lymphoproliferative responses in IRIS patients. **Data depicted are before immunotherapy (solid bars), 4 weeks after IL-2 administration (crossed bars) and 4 weeks after IL-2 plus GM-CSF (hatched bars). Data are shown as median values with interquartile ranges.

**Figure 2:**
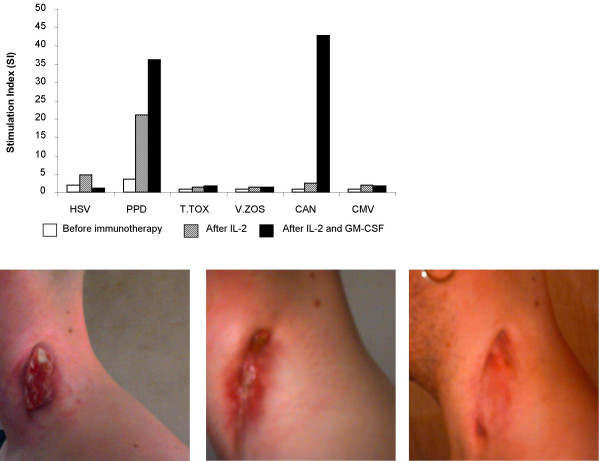
**(a – top) Specific lymphoproliferative responses to recall antigens of patient 4. **Data are at IRIS presentation (white bars), 4 weeks after IL-2 administration (hatched bars) and 4 weeks post final IL-2 and GM-CSF dosing (solid bars). Photographs depict the clinical manifestation of MAC lymphadenitis of the neck in patient 4, at IRIS presentation **(b – bottom left)**, 4 weeks after IL-2 administration **(c- bottom centre)**, and 4 weeks after IL-2 plus GM-CSF administration **(d – bottom right)**.

Immunotherapy was initiated for severely immuno-compromised patients with exacerbated underlying MAC infection (IRIS) who were unable to achieve remission after receiving ART and anti-mycobacterial therapy. Four patients with median CD4^+ ^T-cell counts of 70 cells/μl (IQR 63–123) after a median 10 months on ART, with persistent MAC infection, received IL-2 and GM-CSF (see Table 1 for drug regimen). PPD-specific T-cell responses and responses to other recall/viral antigens were absent in all patients before immunotherapy (Fig 1a). After administration of immunotherapy, we saw an increase in median CD4^+ ^T-cell counts to 171 cells/μl (IQR 128–302) (Table 1). Moreover, we observed robust antigen-specific T-cell responses to a panel of antigens including PPD. Such responses were sometimes more vigorous than those observed in patients on ART alone (Fig 1a), and were paralleled by remission from the underlying MAC infection. Furthermore, immune reconstitution characterised by a rise in CD4 T-cell counts and constant undetectable plasma viremia was achieved. Patient 4 was admitted with localised MAC associated lymphadenitis of the neck and lacked *in vitro *proliferative responses to PPD and other recall antigens despite a CD4 T-cell count of 280 cells/l μblood. After administration of immunotherapy we observed a rapid recovery in immune function (Fig 2a). The parallel clinical manifestations depicted an improvement in the neck lesion after IL-2 therapy and complete remission post administration of IL-2 plus GM-CSF (Fig 2b–d). We also observed an increase in CD4 T-cell counts from 280 to 601 cells/μl during this period (Table 1). No significant changes were seen in HIV-1-specific T-cell responses, which remained undetectable throughout the study (Fig 1b).

**Figure 3:**
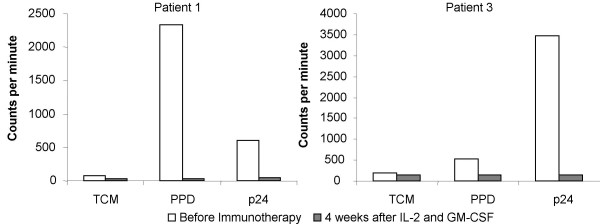
**IL-4 production in culture supernatants of PBMC from 2 patients. **Data are from 5 days stimulation with/without PPD and p24 antigens, before immunotherapy (white bars) and 4 weeks after administration of IL-2 plus GM-CSF (hatched bars). Data are expressed as the mean cpm (proliferation of cell line CT.h4S) for triplicate cultures, with an error of the mean of ± 15%.

In 3/4 IRIS patients we carried out IL-4 bioassays in culture supernatants, rather than ELISA, in order to assess the levels of bioactive cytokine being produced. We were able to detect production of IL-4 in cultures with antigens to which the patients had been previously exposed including anti-PPD responses in 2/3 patients (Fig 3). Upon initiation of immunotherapy there was a decrease in IL-4 production, which was paralleled by restoration of proliferative specific-anti-PPD T-cell responses.

### Lymphoproliferative T-cell responses to recall/viral antigens in asymptomatic clinical progressors and seronegative controls

**Figure 4:**
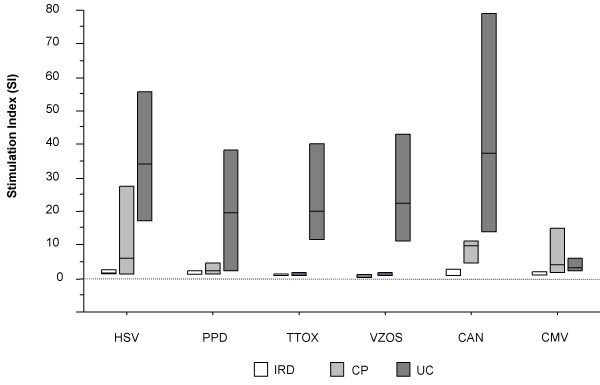
**Box-plots depicting specific lymphoproliferative responses to different recall antigens, during manifestation of IRIS and comparison with clinical progressors with no IRIS and uninfected controls. **The antigens used are shown on the x-axis. Bars denote median responses with interquartile ranges. White boxplots represent IRIS patients receiving ART and immunotherapy. Non-IRIS patients are depicted by dotted boxplots and uninfected controls by hatched boxplots.

We assessed T-cell proliferation in CP and uninfected controls (UC) and compared these with the responses seen in IRIS patients. CP presented a median 3.9 fold increase in CD4 T-cell counts 6 months post initiation of ART from 76 cells/μl (IQR 22.5–90) to 249 cells/μl (IQR 187.5–303.5) (*p *< 0.001) (Table 1). These patients remained clinically asymptomatic and had detectable specific T-cell responses to at least one recall antigen (Fig 4). All UC showed vigorous responses to recall antigens (Fig 4).

### Flow cytometry revealed higher levels of CD38 and HLA-DR expression and lower levels of naïve CD4 T cells in IRIS patients than in asymptomatic clinical progressors

Compared to CP, IRIS patients showed significantly higher percentages of CD4^+^HLA-DR^+ ^T lymphocytes (*p *< 0.005), and significantly higher percentages of CD8^+^CD38^+ ^T cells (*p *< 0.05) (Table 2). When activation was quantified on a per cell basis, IRIS patients showed higher levels of activation of both CD4 and CD8 T cells compared to CP (*p *< 0.02 and *p *< 0.01, respectively), as demonstrated by analysing the mean fluorescent intensity levels of CD38 expression (Table 2). Furthermore, the median percentage of naïve CD4^+^CD45RA^+^CD27^+ ^T cells in IRIS patients was significantly lower than in CP and UC (*p *< 0.005). These observations are not surprising as IRIS patients were more immuno-compromised when therapy was initiated suggesting that IRIS may be associated with persistent hyperactivation of both CD4 and CD8 T lymphocytes, and associated with a lack of naïve CD4 T cells possibly due to absence of thymic function.

**Table 2:** Percentages of different lymphocyte subsets in the CD4^+ ^and CD8^+ ^T cell population, in immune reconstitution inflammatory syndrome patients, clinical progressors and uninfected controls.

	CD4	CD8	
		
	IRIS		*p *value
HLA-DR	27.4 (16–38.4)	28.7 (24.6–32)	< 0.005/n.s
CD38	77 (70–78.5)	82.6 (75.3–90.7)	n.s/< 0.05
CD38 MFI	1118 (1253-780)	493 (548-297)	< 0.02/< 0.01
Naïve	19.4 (6.9–23.9)	25 (7–42)	< 0.005/n.s
Memory	85 (44–96)	53.8 (37.5–76)	n.s/n.s
			
	CP		
			
HLA-DR	7.3 (5.4–9.2)	30.5 (22–40)	
CD38	66 (60.7–71.7)	73 (61.7–80)	
CD38 MFI	173 (85–211)	133 (105–146)	
Naïve	35 (30–45)	32 (24.6–42)	
Memory	59 (54–67)	38 (26–46)	
	UC		
HLA-DR	4 (2.8–5.3)	7.6 (6.9–9.9)	
CD38	70 (68–75.5)	66 (66–70)	
CD38 MFI	97 (71–116)	85 (58–87)	
Naïve	40.8 (40–49)	60.4 (51.2–61.5)	
Memory	30 (29.2–40)	22 (20.8–30.3)	

IRIS- Immune reconstitution inflammatory syndrome patients; CP- asymptomatic clinical progressors; UC- uninfected controls. Data are shown as median percentage, and CD38 mean fluorescent intensity (MFI) (interquartile range). *p *values shown between IRIS and CP patients for CD4/CD8 data respectively.

## Discussion

Immune reconstitution after initiation of ART may be concurrent with both an increase in immuno-pathological responses against opportunistic pathogens and with the induction of IRIS [11-19,24]. The IRIS phenomenon has been ascribed to vigorous immune responses specific to underlying pathogens, with clinical manifestations related to the immune response elicited against such pathogens. Typically, IRIS patients have an undetectable viral load, and CD4 T-cell counts that have rapidly increased, by 3 or 4 fold, shortly after initiation of ART. Previous studies have used delayed type hypersensitivity (DTH) tests to assess the cell-mediated immunity of these patients [12,14,15]. In contrast, we used the thymidine incorporation assay to evaluate lymphocyte proliferation. This allows visualisation of *in vitro *immune function of T lymphocytes in peripheral blood and direct comparison with asymptomatic HIV-1^+ ^subjects as well as uninfected controls. Some reports have shown the lack of correlation between these two assays [25], as functionally T-cell proliferation and DTH responses can diverge [26]. Therefore, by utilising the thymidine incorporation assay, we demonstrate the correlation between *in vitro *functional data and clinical evidence.

There were two important findings in this study. Firstly, patients admitted with IRIS lacked antigen-specific T-cell responses. Secondly, administration of IL-2 and GM-CSF appeared to rapidly introduce these responses and was associated with clinical recovery in patients with advanced HIV-1 infection.

Data from uninfected controls and treated asymptomatic clinical progressors, revealed the presence of lymphoproliferative responses to recall/viral antigens, compared to IRIS patients, confirming that functional specific T lymphocytes are associated with the control of opportunistic infections. Thus, the clinical picture presented by our cohort of IRIS patients is likely to be associated with the lack of lymphoproliferation and IL-2 production rather than with robust antigen-specific T cell responses. This suggests an alternative/additional mechanism for IRIS, distinct from previous hypotheses which suggest that IRIS is caused by pathogen-specific responses induced by successful ART.

Of note, are the generally weak proliferative responses observed in response to other pathogens such as CMV, which recover after initiation of ART [27,28]. Although complete immune impairment appears to be restricted to HIV-1 and PPD antigens, other studies have reported CMV-specific responses to remain generally unchanged in HIV-1+ patients, regardless of the patients' HIV viral loads and clinical state [29]. Different reasons may explain this observation: CMV viraemia is often very low or undetectable and thus fails to induce robust T cell responses [29]; in addition CMV does not target the antigen presenting cells such as dendritic cells and macrophages hence enabling the uninfected antigen presenting cells to efficiently carry out processing and presentation and to generate specific T-cell responses – unlike mycobacteria and HIV-1 which are both known to target antigen presenting cells. In addition, our group has previously shown that in general, HSV- and CMV-specific T-cell responses appear to be more robust in both CP and LTNP [22,28].

The mechanisms behind IRIS still remain elusive. Through cell-to-cell contact, various complex molecular interactions and the production of cytokines, CD4 T helper lymphocytes modulate the activity of all cells involved in both innate and acquired immunity, including virus-specific cytotoxic CD8 T lymphocytes [30-33]. Although anti-HIV-1 CD8 T cells are present in chronically infected individuals, they lack specific functional properties, most likely because CD4 T cell help is impaired [34-37]. The HIV-1-induced defect of CD4 T-cell responses is likely to be an underlying mechanism causative of anergy and subsequently IRIS, due to defective antigen presentation and lack of T-cell help.

The use of immunotherapy in severely immuno-compromised patients has been shown to have beneficial effects in partially reversing the CD4 T-cell defects exerted by HIV-1, when administered concomitantly with ART [9,21]. Such data concurs with our previous findings that concomitant administration of ART and IL-2 plus GM-CSF reversed the type 2 anti-proliferative cytokine environment, decreasing IL-4 levels and inducing pathogen-specific proliferative responses. We have previously shown that such regimens induce HIV-1-specific proliferative CD4 and IFN-γ secreting CD8 T-cells. In these studies, HIV-1-specific proliferative responses paralleled by increased IL-2 production, responsiveness and up-regulated expression of IL-2-specific mRNA were associated with remission of disease [21]. It is important to note that, in previous studies, GM-CSF was administered at doses twice the level of those described here, thus possibly inducing HIV-1-specific immunity. This may explain our inability to induce detectable HIV-1-specific T-cell responses despite a decline in IL-4 production. Regardless, in our cohort of immunotherapy recipients, remission from MAC occurred rapidly despite profound immuno-suppression.

During HIV-1 infection immune hyperactivation is accompanied by up-regulation of surface expression of CD38 and HLA-DR on CD4 and CD8 T cells [38-41]. Higher T-cell activation in the IRIS cohort compared to CP is not surprising, as T-cell specific responses were much lower in IRIS patients. This is in concordance with data from Caruso *et al *describing the occasional lack of correlation between the percentage of T cells expressing activation markers and thymidine incorporation [42]. It is suggested that hyperactivation by HIV-1 and other underlying pathogens is associated with activation induced cell death and/or anergy [43]. Furthermore, high HLA-DR levels are suggestive of increased T cell↔T cell antigen presentation, which is associated with induction of T-cell anergy/dysfunction and increased IL-4 production [44,45]. This is in agreement with our previous findings that anergised antigen-specific T cells are present at baseline in immunocompromised patients but lack proliferative/functional ability [21]. The lower levels of naïve T cells observed in IRIS patients compared to CP may be due to a more immunocompromised state and possibly reflect thymic dysfunction/inactivity.

Our data suggests that the degree of immune reconstitution achieved with potent ART alone is dependent on the clinical stage of the patient when therapy was initiated. Furthermore, we hypothesise that in some late-stage patients ART may elicit the expansion of abnormal/anergic T cell clones responsible for an erratic immune response. Concomitant administration of IL-2 and GM-CSF may be associated with provision of proliferative signals to immature thymocytes and/or rescue of anergic CD4 T cells to generate fully functional T-cell responses. GM-CSF acts directly on antigen presenting cells, including macrophages, which may induce and contribute to a more rapid remission from intracellular infection [46-48].

## Conclusion

In conclusion, ART induced immune reconstitution restores specific responses, which likely help in the clearance of opportunistic pathogens. However, inadequate immune responses observed in treated late-stage disease, in addition to other possible confounders, may be causative of IRIS. Administration of IL-2 and GM-CSF concomitantly with ART in late-stage patients may result in proliferation of pathogen-specific CD4 T-lymphocytes, which likely enable a more rapid clearance of intracellular pathogens such as *Mycobacterium avium*. Such responses may be associated with a better outcome and, possibly quicker recovery, in immuno-compromised patients who fail to achieve immune reconstitution with ART alone, indicating that this therapeutic approach as salvage immuno-therapy may have an impact on short-term mortality. The small number of patients is noteworthy- this is nonetheless an interesting and provocative finding that deserves further prospective exploration in larger numbers of similar patients.

## Competing interests

The author(s) declare that they have no competing interests.

## Authors' contributions

AP carried out the proliferation assays, phenotypic analysis, data analysis, participated in the study design and wrote the manuscript. MN, ALP, MF and BG recruited patients and participated in the study design. FG participated in the study design and participated in the drafting of the manuscript. NI conceived the study, carried out proliferation assays and bioassays, data analysis and the design, coordination, the draft and finalisation of the manuscript. All authors read and approved the final manuscript.
